# Esketamine may be an ideal substitute for ketamine during cochlear function measurement

**DOI:** 10.1590/1414-431X2021e11503

**Published:** 2021-08-20

**Authors:** Yufeng Li, Xuehua Zhou, Xia Shen

**Affiliations:** 1 Department of Anesthesiology, Eye & ENT Hospital, Fudan University, Shanghai, China

**Keywords:** Esketamine, Ketamine, Anesthesia, Auditory brainstem response, Distortion product otoacoustic emission

## Abstract

The mixture of ketamine and xylazine is widely used for the auditory brainstem response (ABR) measurement. Esketamine is twice as potent as ketamine. Our objective was to assess the influence of esketamine in mice undergoing cochlear function measurement including ABR and distortion product otoacoustic emission (DPOAE) measurement. C57Bl/6J mice were treated with an equivalent dose of analgesia and received either a single intraperitoneal (*ip*) injection of 100 mg/kg ketamine and 25 mg/kg xylazine or 50 mg/kg esketamine and 25 mg/kg xylazine. Hearing thresholds, peak latencies of waves I and V, and DPOAE thresholds were recorded. Time to loss of righting and time to regain righting were also assessed. We found that hearing thresholds, the peak latencies of waves I and V, and DPOAE thresholds were similar between the two groups (all P>0.05). Time to regain righting was significantly shorter in the esketamine group (P<0.001) than in the ketamine group. We concluded that when using equivalent doses of analgesia, esketamine may be an ideal substitute for ketamine during cochlear function test.

## Introduction

The auditory brainstem response (ABR) threshold is commonly used to assess hearing sensitivity and is defined as the lowest sound level that elicits an ABR peak. The ABR threshold is also used to evaluate the integrity and function of the peripheral and proximal auditory systems in animal research. The ABR is an important procedure for diagnosing hearing loss in infants. Clinically, most children require sedation to remain still during ABR test to prevent movement artefact interfering with the ABR trace (1). Given that ABR is generated in the central nervous system and that general anesthesia affects neurotransmission, it is important to evaluate the effects of anesthetic compounds on ABR to appropriately interpret hearing sensitivity.

Distortion product otoacoustic emissions (DPOAE), generated by the cochlea when two simultaneous pure tones (f1 and f2) are emitted, is another recommended method to detect congenital hearing loss in neonatal patients. It is reported that general anesthetic can significantly change DPOAE amplitudes (2).

The widely used general anesthetic ketamine is a non-competitive antagonist of the N-methyl-d-aspartate receptor that has been in clinical use since the 1960s (3). A mixture of ketamine and xylazine is reported to have no effect on ABR amplitude or threshold except for a slight increase in ABR latency (4). Racemic ketamine contains two enantiomers: esketamine (S(+)-ketamine) and arketamine (R(-)-ketamine) (5). Interestingly, the anesthetic effect of esketamine is twice as potent as that of ketamine (6,7). At equivalent doses, esketamine has fewer psychotropic side effects and improves concentration, primary memory, and recovery in healthy volunteers compared with ketamine (8). In this study, we compared the efficacy of ketamine and esketamine during ABR and DPOAE measurement in mice.

## Material and Methods

### Animals and anesthesia

All procedures involving the study animals were approved by the Animal Care and Use Committee of Eye & ENT Hospital, Fudan University and were in accordance with the National Institutes of Health Guide for the Care and Use of Laboratory Animals (Protocol No. 2016/03/11).

The ABR measurements (n=9 in the ketamine group and n=8 in the esketamine group) were performed on C57Bl/6J male mice of 28 to 30 days of age (Shanghai SLAC Laboratory Animal Co., Ltd., China). The experimental protocol and mice used in this study were approved by the Institutional Animal Care and Use Committee of Fudan University.

In the ketamine group, each mouse was anesthetized using *ip* injections containing 100 mg/kg ketamine (50 mg/mL, Gutian Pharmaceutical, China) and 25 mg/kg xylazine (Sigma-Aldrich, China). Because the anesthetic effect of esketamine is twice as potent as ketamine (6,7), each mouse in the esketamine group was anesthetized with *ip* injections containing 50 mg/kg esketamine (25 mg/mL, Hengrui Pharmaceutical, China) and 25 mg/kg xylazine.

### ABR measurement

Hearing thresholds were determined by ABR testing. Prior to testing, we confirmed that each mouse had normal external auditory canals and tympanic membranes by otoscope. Mice were also confirmed to lack tympanitis. Mice were anesthetized with a mixture of either ketamine/xylazine or esketamine/xylazine, and their body temperature was maintained at 37°C using a thermostatic heating pad. ABR testing was performed in a soundproof anechoic chamber, and responses were recorded using subcutaneous needle electrodes positioned at the right mastoid prominence, cranial vertex, and nose tip. An analog-to-digital converter (AD3; Tucker-Davis Technologies, USA) was used to amplify (100,000 U), filter (0.3-3.0 kHz), and digitize neuronal activity. Tone burst stimuli (5 ms duration with 0.5 ms rise-fall) were emitted at four frequencies (8, 16, 24, and 32 kHz) from a speaker placed 5 cm from each mouse. The mean response to 1,000 repetitive stimuli was recorded for each animal. Hearing thresholds were determined for each frequency by systematically decreasing the intensity of the stimuli. Specifically, intensities were decreased from 100 dB sound pressure level (SPL) to 20 dB SPL in increments of 10 dB SPL. Below 20 dB SPL, the increment was reduced to 5 dB until a recognizable wave response (two or more consistent characteristic waveforms) was noted.

### DPOAE measurement

DPOAE was measured with a TDT-RZ6 system (Tucker-Davis Technologies). The stimuli to elicit DPOAEs were two sine wave tones of differing frequencies (F2=1.2F1) with 1 s duration and F2 ranging from 4 to 40 kHz. The two tones were given at identical intensities, which ranged from 20 to 90 dB SPL with 10 dB increments. The acoustic signal was digitized at 200 kHz and the magnitude of the 2F1∼F2 distortion product was determined by Fast Fourier Transformation (FFT). Surrounding noise floor was also determined by averaging 20 adjacent frequency bins around the distortion product frequency. DPOAE thresholds were determined offline by interpolating the data and identifying when the signal was over −5 dB SPL and over two standard deviations above the noise floor. If no DPOAE signal was detected even at our equipment limits of 90 dB SPL, the threshold was arbitrarily defined to be 90 dB.

### Statistics

We have previously reported the ABR threshold in ketamine-treated mice to be 28.1±5.1 dB at 8 kHz (9). Based on an estimated 25% change in ABR thresholds, we determined that each group required at least 8 mice to produce a statistical power of 80% at a 5% level of significance in our study.

All data are reported as means±SE. Normal distribution of the data was confirmed with the Shapiro-Wilk test. Two-way repeated measures analysis of variance (ANOVA) was used to analyze differences in hearing function (i.e., ABR thresholds, peak latencies, and DPOAE thresholds) between the ketamine and esketamine groups. We used Student's *t*-tests to compare time to loss of righting and time to regain righting. Significance was defined at a P value of 0.05. All statistical analyses were performed using SAS software version 9.2 (SAS Institute Inc., USA).

## Results

### Esketamine did not inflate ABR hearing thresholds

**Figure 1:**
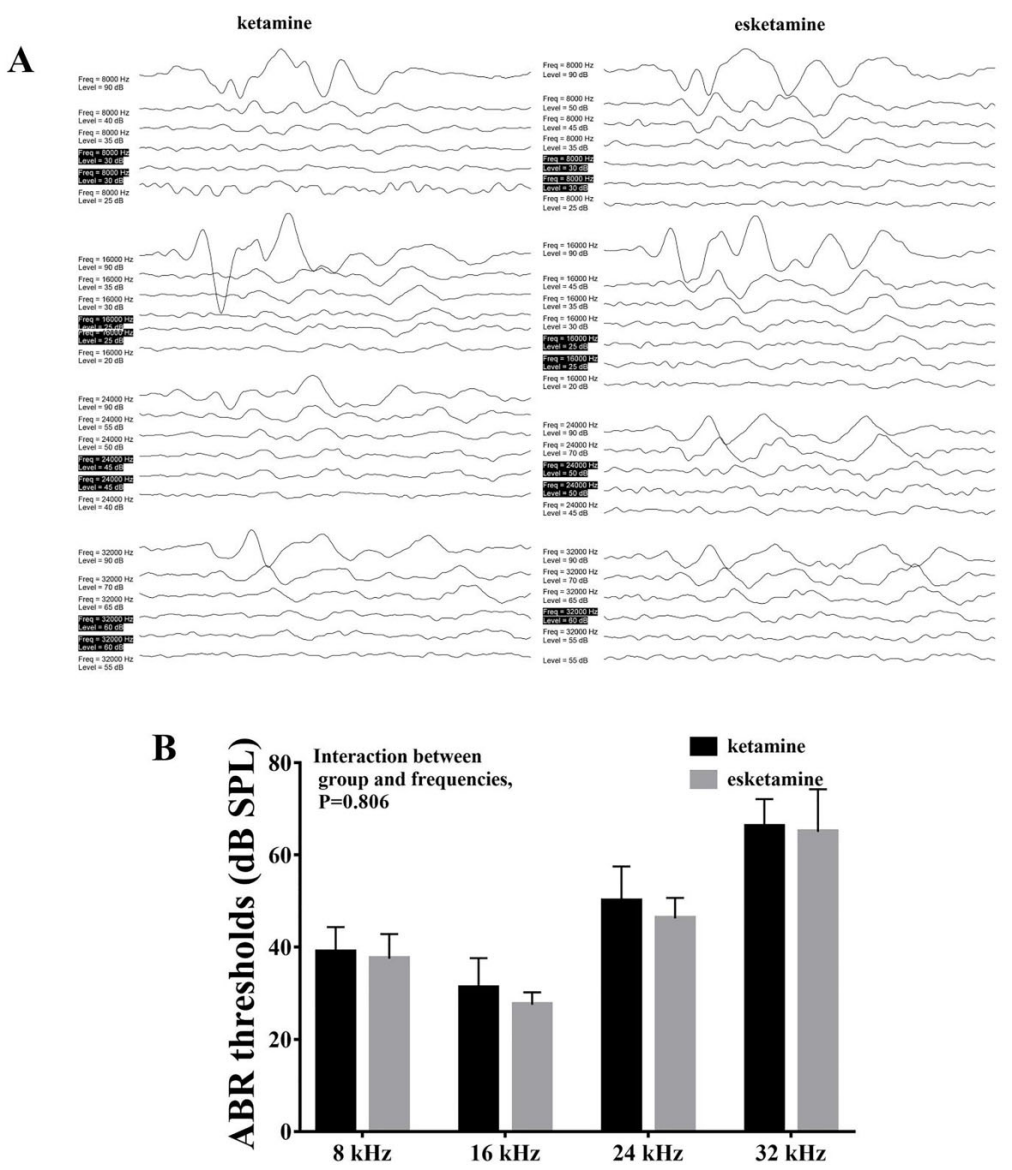
Esketamine did not increase auditory brainstem response (ABR) hearing thresholds in mice. **A**, ABR measurement at four different frequencies between the ketamine and esketamine groups. **B**, Hearing thresholds were not increased by esketamine at 8, 16, 24, and 32 kHz frequencies. Data are reported as means±SE. ANOVA was used to compare the groups. SPL: sound pressure level.

To evaluate hearing function, we measured the ABR thresholds of mice in the ketamine and esketamine groups at four frequencies (8, 16, 24, and 32 kHz) (Figure 1A). Statistical analysis using ANOVA did not show a significant difference in hearing thresholds over the four frequencies between the ketamine and esketamine groups (P=0.806) (Figure 1B).

### Esketamine did not affect nerve fiber recruitment

**Figure 2:**
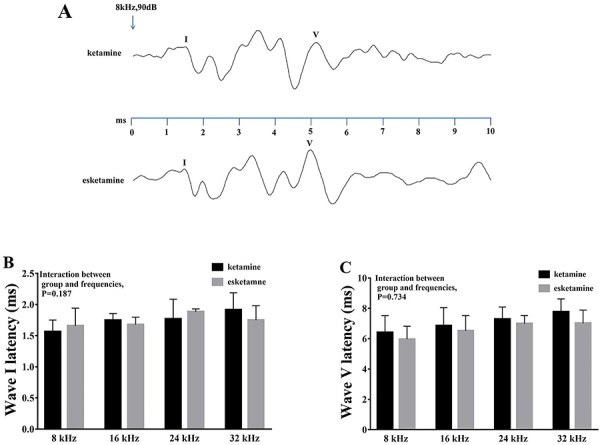
Esketamine did not affect nerve fiber recruitment in mice. **A**, Peak latencies of waves I and V with a stimulus of 8 kHz and 90 dB sound pressure level. **B** and **C**, Esketamine did not reduce the peak latencies of waves I or V at the four frequencies assessed. Data are reported as means±SE. ANOVA was used to compare the groups.

We calculated the peak latencies of waves I and V with a stimulus of 8 kHz and 90 dB SPL (Figure 2A). Statistical analysis using ANOVA did not show a significant difference in peak latencies of wave I over the four frequencies between the ketamine and esketamine groups (P=0.187) (Figure 2B). Likewise, ANOVA did not show a significant difference in peak latencies of wave V over the four frequencies between the ketamine and esketamine groups (P=0.734) (Figure 2C).

### Esketamine did not affect outer hair cell function

**Figure 3:**
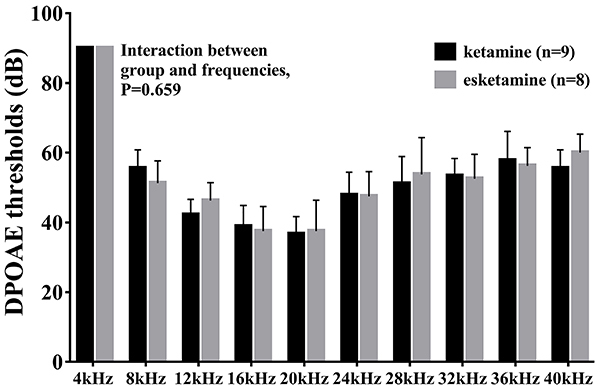
Esketamine did not affect outer hair cell function in mice. Distortion product otoacoustic emission (DPOAE) measurement showed that the function of outer hair cells was similar between the ketamine and esketamine groups. Data are reported as means±SE. ANOVA was used to compare the groups.

To evaluate the anesthesia action on outer hair cell function, DPOAE thresholds from 4 to 40 kHz sound stimuli were measured. Similar to the findings for ABR, statistical analysis using ANOVA did not show a significant difference in DPOAE thresholds at different frequencies between the ketamine and esketamine groups (P=0.659) (Figure 3).

### Esketamine provided fast recovery

All ABR measurements in mice were completed after a single *ip* injection of anesthetic and without obvious adverse events. After *ip* injection of ketamine or esketamine, the mean time to loss of righting was 75.9±19.2 s in the ketamine group and 61.6±26.6 s in the esketamine group. Although a trend suggested that esketamine took effect faster than ketamine, this was not statistically significant (P=0.08). In contrast, the time to regain righting was 117.9±25.9 min in the ketamine group and 47.1±5.8 min in the esketamine group (P<0.0001).

## Discussion

Our results indicated that, compared with ketamine/xylazine anesthesia, the use of esketamine/xylazine anesthesia did not significantly affect hearing thresholds and nerve fiber recruitment during ABR measurement. Esketamine anesthesia also did not affect DPOAE thresholds. Nonetheless, the use of esketamine seemingly provided faster recovery in mice compared with the use of ketamine.

ABR is commonly used in laboratory animals to evaluate hearing sensitivity. As animals must be immobilized to perform ABR, general anesthetics, such as isoflurane and ketamine/xylazine, are necessary (9-11). Isoflurane is an inhalation anesthetic that is widely used in audiometry to prolong immobilization in small rodent models. However, isoflurane anesthesia is reported to substantially increase ABR thresholds compared to ketamine/xylazine anesthesia, and differences in hearing sensitivity detected under isoflurane may reflect an isoflurane-dependent effect (10,11).

Ketamine and xylazine combinations are also common in animal research. Ketamine confers stable and low ABR thresholds, which is an important advantage in hearing research (4,11). Esketamine is a split of (R, S) ketamine that is also used as an anesthetic drug in several countries (5). In terms of anesthetic effect, esketamine is twice as potent as ketamine. In our study, using an equivalent dose of analgesia in mice, we found that esketamine anesthesia did not affect ABR hearing thresholds, which suggested that esketamine did not elevate hearing sensitivity compared to ketamine. In addition, the peak latencies of waves I and V were similar between the two groups, thus suggesting that esketamine did not impair nerve fiber recruitment.

DPOAE measurement is a useful tool to quantify the contribution of the ‘cochlear amplifier’, mediated by outer hair cell electromotility (12,13), which is a principal factor driving inner hair cell sound transduction. In the current study, mice in the esketamine group showed a similarity in DPOAE thresholds compared to the ketamine group. Sheppard et al. (2) reported that ketamine/xylazine has minor effects on DPOAE amplitudes in rats when compared with isoflurane. Our findings suggested that the effect of esketamine anesthesia on the function of outer hair cells was as minor as ketamine anesthesia.

We found that a single dose of a mixture of xylazine with either esketamine (50 mg/kg) or ketamine (100 mg/kg) was both safe and well-tolerated in mice without the onset of serious adverse events. The mean time to loss of righting was shorter using esketamine; however, this difference was not statistically significant. Such a result may be due to the small sample size used in our study. Moreover, the time to regain righting was significantly shorter in the esketamine group. Our findings are consistent with the results of previous studies in humans demonstrating that esketamine, when compared with ketamine, resulted in a shorter recovery (9 *vs* 13 min, P<0.05) and orientation recovery times (11.5 *vs* 17 min, P<0.05) after a short period of anesthesia (14). A quicker clearance of esketamine than R-ketamine (14) may explain the faster recovery observed following the use of esketamine anesthesia.

A fast recovery period has multiple clinical advantages. ABR measurements are the gold standard for diagnosing hearing impairments in infants and young children and require the use of anesthesia (1). Ideally, a sedative would have rapid onset, provide a sufficient level and duration of sedation, and allow for a rapid recovery. Ketamine is also combined with propofol during pediatric sedation for ABR testing (15) and brief painful procedures (16). Considering the efficacy of esketamine in ABR testing and the fast recovery of the mice in our study, esketamine may be used to better meet the requirements necessary to complete brief procedures, such as the ABR procedure in children who require sedation. However, further studies are warranted in a pediatric setting to evaluate the dose titration, efficacy, and safety of esketamine for this practice.

In conclusion, the use of esketamine as an anesthetic did not impair cochlear function and provided faster recovery in mice compared with the use of ketamine anesthetic. Therefore, esketamine is an attractive option for use as a general anesthetic during brief procedures.
